# Notes and News

**Published:** 1887-07-30

**Authors:** 


### Notes and News.

Collected and Communicated.

Miss Simpson, of Harrogate, has been unanimously appointed matron to the Blackburn and East Lancashire Infirmary. Miss Simpson has had fifteen years' experience in hospital work at Coventry, Oldham, and Birmingham.

A *fête* was held on behalf of the Herts Convalescent Home at St. Leonards on Thursday, July 28th, to which an official representative of every sick and benefit club in Hertfordshire was invited.

The new governing body of the Aberdeen Royal Infirmary created by Act of Parliament, held its first meeting in the Town and County Hall on Tuesday afternoon, in terms of the said Act. Baillie Kinghorn, in the absence of the Lord Provost Henderson, presided, and there was a good attendance.

Mrs. Sturt, of Sonnenburg Park Hill, who takes an untiring interest in the Children's Convalescent Home at Croydon, says, in her survey of the year's work : "In forwarding to you the report of the seventh year of the Children's Convalescent Home, I have much pleasure in stating, and cause for great thankfulness, that it has been one of renewed prosperity; and although several bad cases have been received, all have happily benefited. I could state several cases showing how quickly good food and fresh air tell upon the little ones."

In consequence of the continued scarcity of work among two industrial classes of the community, the committee of the West Bromwich Self-Supporting Dispensary resolved at their monthly meeting on Tuesday to admit suitable persons to the benefits of the Dispensary until the end of the year without an entrance-fee.

The *Echo* says :—" In these days when economy in the management of charities is of the first importance, it is well to note that the authorities of the Westminster Hospital find that a cot in their new children's wing can be permanently endowed for £600. In nearly all other London children's hospitals the endowment sum is fixed at £1,000."

At Burns's town of Ayr the Committee of the Ayr Charity Cup have, through the hands of Mr. William Auld, this year handed over to the treasurer of the County Hospital the handsome sum of £48 0*s*. 11*d*. This is the third year since the Charity Cup Competitions were instituted.

Sir A. G. Hazelrigg, Bart., presided at the quarterly meeting of the Governors of the Leicester Infirmary on Tuesday. Mr. T. F. Johnson, J.P., reported that at the close of the quarter the balance due to the treasurer was £598 0*s*. 5*d*. This item was not considered unsatisfactory, seeing that the adverse balance at the same period last year was £1,500. A hope was expressed that this amount might be still further reduced, and that in due time there might be a satisfactory balance on the credit side.

At the quarterly meeting of the Governors of the Norfolk and Norwich Hospital, held on Saturday, Lieutenant-Colonel Mansel presiding, sundry improvements, especially in the gardens and grounds, were proposed and agreed to. Sir Peter Eade, M.D., supporting the motion, spoke of the decidedly advantageous effect which bright gardens and well-kept grounds had upon those patients who were returning to convalescence.

The quarterly meeting of the trustees of the Newton Cottage Hospital, Massachusetts, was held on the 20th June last, when, in consequence of the report of the building committee, it was decided to direct the finance committee to obtain funds for the erection of an additional ward. The report stated that the need of this ward was steadily growing more imperative, as there was not sufficient accommodation for the women patients who applied for relief. We have pleasure in quoting from the report the following eloquent testimony to the efficiency of the new matron, Miss Mary F. Palmer : "Her conduct of the hospital during the last month appears to the committee amply to justify their confidence, and to be a proper subject of congratulation." It is indeed a subject of congratulation, and a matter for deep satisfaction on the part of hospital authorities, when they have succeeded in securing the services of a thoroughly capable and trustworthy matron, for upon her exertions depend to a very large extent the comfort of the inmates and the usefulness of the institution.

THE following vacancies are announced this week, the amount of the salary being placed in each case after the name of the institution :—

**Resident Medical Officers.**

Belgrave Hospital for Children. House Surgeon.—£20.
Carlisle Dispensary. Junior House Surgeon.—£100.
King's Lynn Hospital. House Surgeon and Secretary. Doubly-qualified.—£80.

**Non-resident Medical Officer.**

Lampeter Union. Medical Officer and Vaccinator. Registered.—£60.

**Trained Nurses.**

Batley Cottage Hospital. One.—£20.
Ipswich Hospital. Several.
Ipswich Hospital. One for Children's Wing.—£25.
Lowestoft Hospital. One Night Nurse.—£20.
Newark Hospital. One for private work.
Newcastle, St. Mary Magdalene Home. One.—£20.
Nottingham Nursing Institution.—Several.
West Derby Union. One.—£20. Apply, Matron, Mill Road Infirmary, Everton, Liverpool.

---

THE managers of the Cork Dispensary are evidently under the influence of that kind of laziness which usually results from excessive heat. The fortnightly meeting of the committee should have been held a few days ago at the Dispensary, Liberty Street, at 12 o'clock, but no member putting in an appearance, no business could be done. Perhaps at the next fortnightly meeting the thermometer may give a more moderate register, and some of the members may be sufficiently recovered to attend.

---

A SHORT time since, it was stated that Mr. and Mrs. Kendal had kindly consented to give an entertainment in aid of the funds of the Children's Hospital, Birmingham. It was hoped that the entertainment would have taken place before this, but owing to the successful and extended season at St. James's Theatre, Mr. and Mrs. Kendal have been unable to leave London, and the promised entertainment has now to be postponed till a more convenient time.

---

THE United Friendly Societies of Bournemouth are about to make a special effort on behalf of the local medical charities. A meeting was recently held under the presidency of Mr. H. A. Garrett, and arrangements were made for a demonstration of the Societies on Hospital Sunday. A hope was expressed that ladies would come forward, and render assistance in the matter.

---

THANKS to the kind thoughtfulness of the Board of Management of the Jenny Lind Infirmary at Norwich, the nurses and medical staff were on Wednesday and Thursday the recipients of a very enjoyable treat in the shape of a river excursion. The *City of Norwich* steamer was chartered for the purpose, and the senior surgeon, W. Cadge, Esq., placed his yacht at the service of the chairman and members of the Board of Management and medical staff, who with their wives joined the holiday party in the cruise. The enjoyment of the visitors was greatly enhanced by the kindness of Dr. and Mrs. Beverley, who invited them on their return journey to anchor at Brundal, and take tea in the host's grounds on the banks of the Yare.

ON Sunday afternoon a musical concert in aid of Leeds Infirmary and the Clayton Hospital at Wakefield was given in a field at Altofts, near Normanton, under the auspices of a committee representing fifteen Friendly Societies of Normanton and district. There were five brass bands in attendance, which paraded the town previous to the concert, making collections *en route*. The musical service consisted of the National Anthem, selections from the "Messiah," "Judas Maccabæus," the "Creation," and the "12th Mass." There were about two hundred performers, and Mr. J. Copeland conducted. Dr. Mackenzie, who presided, described the proceedings as a religious ceremony. Large numbers of the public were present, and contributed liberally to the funds.

---

THE annual meeting of the subscribers to the Clayton Hospital and Wakefield Dispensary was held at the hospital on Wednesday. In the absence of Mr. Percy Tew, J.P., the President, the Rev. Norman Stratton, the Vicar, presided, and there were also present Dr. Holdsworth, J.P., Col. Mackie, J.P., Alderman W. H. Lee, J.P., the Revs. H. G. Parish, W. H. Dyson, R. Westrop, — Binks, Hon. Sec., and others. The vicar congratulated the friends of the institution on its having reached its hundredth anniversary. He much regretted that the congregational collections and annual subscriptions showed a falling off. It was evident, he said, that there had been great economy in the management of the institution, inasmuch as the diminished income had been so used as to fully meet the expenditure. Special thanks were given to the working men of the district for their earnest and successful efforts on behalf of the funds of the charity.

---

THE annual meeting of the governors and subscribers of the Great Northern Central Hospital was held on Friday, Mr. C. T. Murdoch, M.P., presiding. During the year £7,899 had been subscribed to the building fund, and £8,230 paid to the contractors, leaving a balance in hand of £1,969. The committee were glad to be able to say that at the present time there was a movement on foot in the parish of Islington to raise the money for a Jubilee memorial, and that its promoters had decided that it should be devoted to the building of a Jubilee wing to the new hospital. It is hoped that this wing will be opened about the beginning of November. The secretary read a telegram from the treasurer of the Hornsey Children's Jubilee Fund, announcing that the surplus of the fund, amounting to nearly £60, would be paid to the building account of the hospital.

---

THE Grand Bazaar which has been long anticipated on behalf of the Lincoln County Hospital was opened on Thursday, in the west wing, by Mrs. Sibthorp. There were eight stalls, presided over respectively by Lady Mary Turner, Mrs. Clements, and Mrs. Alfred Burton, the Hon. Mrs. Leslie-Melville and Mrs. Hutton, Mrs. Sibthorp, Mrs. H. W. Hutton, Mrs. Borradaile, and Miss Bromhead, the Misses Leslie-Melville, Mrs. Mitchenson, Mrs. Harrison, Mrs. Lowe, Mrs. Brook, and Mrs. Wilkenson. The children's stall was under the superintendence of Mrs. Kerant and Mrs. Charles Brook.

At Plymouth, by permission of the committee of the Royal Albert Hospital, two open air-concerts were given in the afternoon and evening in the grounds of that institution by the Devonport Choral Society. The band of the Duke of Cornwall's Light Infantry and several soloists were present. The arrangements as to the concert were entrusted to the sub-committee of the Jubilee Celebration Committee, consisting of the mayor, the ex-mayor, and other gentlemen of the locality.

---

A movement has been inaugurated for providing a Convalescent Seaside Home for sick and wounded members of the metropolitan police force. At this particular moment the police are by no means the most popular of public servants, but the unpopularity, we are quite sure, is of a temporary character. There can be no doubt of the urgent necessity for such an institution as that now initiated, though it seems hardly desirable to launch it on the waters of public charity. Could not the members of the police force, by a little co-operation and foresight, provide for themselves all they need in this direction?

---

At Rutherglen, near Glasgow, a public meeting was held in the town-hall buildings, for the purpose of taking steps to raise a fund in the borough towards the building of the proposed new infirmary on the south side of the city. Provost Mitchell presided, and there were present several clergymen, town councillors, and other principal inhabitants. It was moved, seconded, and unanimously carried that this meeting heartily approves of the proposal to erect the Victoria Infirmary in the Queen's Park, and adopts the scheme as one deserving the sympathy and generous support of the royal borough of Rutherglen. Glasgow apparently is to have a third hospital.

---

The Chairman of the Bristol Royal Infirmary, at the quarterly meeting of the Board recently held, stated that the finances of the institution were not in a very flourishing condition. In consequence partly of the extra calls upon their friends for the various movements connected with the Queen's Jubilee, and also from the still depressed condition of trade, the extra donations of the current year had been very few. Hospitals generally throughout the kingdom hoped that the year of Jubilee would have been a year of plenty for them. There are indications in various quarters that it may prove to be rather a year of famine.

---

A new block has been added to the Consumption Hospital at Ventnor. The Marquis of Lorne laid the foundation stone on behalf of her Majesty the Queen. By the bequest of Mr. John Jones, the board of management have received funds to produce an income of £2,000 a year, and it is owing to this gentleman's munificence, who resided in Piccadilly, that the new block has been erected. The architect is Mr. Hellyer, of Ryde, and the builders Messrs. Ingram and Son, of Ventnor. The new block is larger than any of its predecessors, and has a very substantial appearance. The sewerage arrangements for the entire set of buildings are cut off entirely from the town drainage, and are said to be most complete.

At a meeting of the Committee of Management of the City of London Lying-in Hospital, held on Wednesday last, the medical officer in his Monthly Report drew attention to the very satisfactory results achieved in the hospital during the past twelve months. Since that time there had been 331 deliveries in the hospital without a maternal death, although several cases had caused considerable anxiety. There had not been a single case of septicæmia. This he considered most gratifying, as such good fortune had not attended the hospital for many years. The last death occurred early in May 1886.

---

On Thursday a special meeting of the governors of the Newcastle Infirmary was held to consider, and, if approved, pass the resolutions to which the sub-committee came in regard to the management of the hospital. Mr. W. D. Stephens presided. Mr. Amyot moved that the meeting be adjourned for a fortnight, as the requisite ten days' notice had not been given. Mr. A. G. Hedley seconded on other grounds. He said sufficient time had not been afforded the governors to look through the rules; besides, there were not twenty-one governors present to consider them. The chairman said Mr. Rutherford only got the rules from the printers on Wednesday, which was another argument for postponement of that meeting. The resolution was then agreed to, and the special court adjourned.

---

At the Greenwich Jubilee Banquet held at the Miller Hospital, the Rev. D. Reith, Vicar of Christ Church, said he remembered the opening of the Miller Memorial Hospital, and he believed it was their present noble chairman who then made the remark that there was no sectarianism in suffering, and it was a gratifying sight to see ministers of religion of all denominations united together in furtherance of the Hospital Sunday movement. Speaking personally for himself as a clergyman, he would say that the older they got the more were they attached to their own particular form of worship, but he also felt that it did not matter which of the half-dozen roads they got to heaven by, so long as they got there. Referring to the Hospital Sunday movement, he said something was wanted to render it more practical than it was at present. A very large number did not go to church at all, and last Hospital Sunday he tried to reach some of them, and after the morning service got his choir to go round to some of the poorer districts, for a contribution from the inhabitants. Twenty-four of the choir went out with missionary boxes, from two till five o'clock, and returned with an interesting report of their experience. The largest sum given by one person was a shilling, and the good lady who gave it asked for change; not being able to obtain it, she went upstairs in search of a smaller coin, but unsuccessfully, and sadly dropped the coin in the box, with the reflection that she would never see its smiling face again. The other coins consisted almost entirely of coppers, but good work was done when by the experiment they raised £5 for the Hospital Sunday Fund. They wanted, however, a more systematic plan than the present, but he intended to follow the same course, if spared, until next Hospital Sunday.

